# Exploring glomeruli and renal tubules transcriptomic data: Crucial role of the AASS gene in membranous nephropathy

**DOI:** 10.1002/ctm2.70317

**Published:** 2025-04-23

**Authors:** Congcong Jiao, Yuxin Zhao, Yang Shao, Haoshen Feng, Cong Ma, Xiangnan Hao, Xiaomei Liu, Junjun Luan, Xu Yang, Hua Zhou

**Affiliations:** ^1^ Department of Nephrology Shengjing Hospital of China Medical University Shenyang China; ^2^ Department of Rehabilitation Medicine First Hospital of China Medical University Shenyang China; ^3^ Department of Pulmonary and Critical Care Medicine Shengjing Hospital of China Medical University Shenyang China; ^4^ Pathology Room Department of Nephrology Shengjing Hospital of China Medical University Shenyang China

1

Dear Editor,

Membranous nephropathy (MN) is one of the most prevalent causes of nephrotic syndrome. Approximately 80% of patients with MN are diagnosed as primary, which is associated with M‐type phospholipase A2 receptor (PLA2R). The remaining cases are considered secondary MN, whose underlying causes including infections, tumors, autoimmune diseases, or the usage of certain medications.^1^ The pathophysiological mechanisms of MN are intricate and continue to be investigated. We utilised machine learning and experimental validation to analyse glomeruli and renal tubules transcriptomic data to identify key genes associated with MN, focusing on their expressions and underlying mechanisms in MN.

A preliminary screening of target genes was conducted using transcriptome data from glomeruli and renal tubules (Figure 1A), retrieved from the GEO database (Table S1). The datasets for glomeruli (GSE108109, GSE180393, GSE200828) and renal tubules (GSE108112, GSE180394, GSE200818) were merged and adjusted using the sva package. Differential analysis (|log_2_FC| > 1, *P *< 0.05) identified 416 differentially expressed genes (DEGs) in glomeruli and 81 DEGs in renal tubules, with 37 intersecting DEGs obtained from both datasets (Figure 1B,C). Enrichment analyses [Gene ontology (GO), Kyoto encyclopedia of genes and genomes (KEGG), and Metascape] revealed that intersecting DEGs are involved in gas transport, cytochrome P450, and drug metabolism (Figure 1D–F), with cytochrome P450 playing a critical role in the metabolism of various medications.^2^ Understanding the expression changes of these genes may provide insights into the drug‐processing capabilities of kidneys.

**FIGURE 1:**
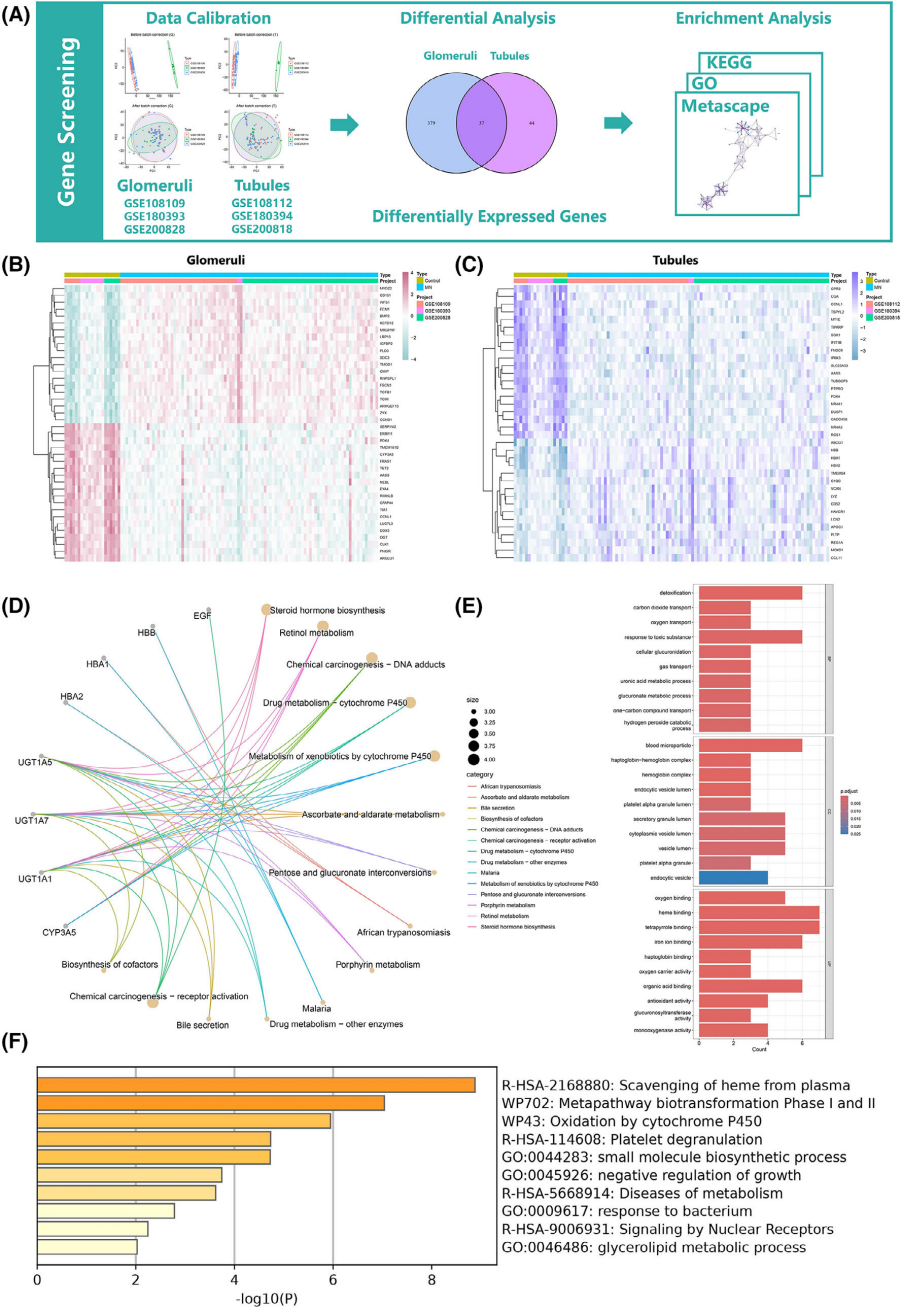
Difference analysis and enrichment analysis results for intersecting DEGs. (A) The flowchart of this part. (B) Heatmap of differential analysis for glomeruli data. (C) Heatmap of differential analysis for renal tubule data. (D) Circled line graph of KEGG. (E) Bar chart of GO enrichment results. (F) Bar chart of Metascape enrichment results. DEG, differentially expressed genes.

We employed random forest (RF) and support vector machine (SVM) to improve the prediction accuracy, thereby identifying key genes in MN (Figure 2A, Figure S1). By employing root mean square error analysis, the 10 most significant genes were determined (Figure 2B, C), leading to the identification of AASS, CYP3A5, IP6K3, and PDK4 in glomeruli data (Figure 2D), while AASS and MT1A were highlighted in renal tubular analysis (Figure 2E). Differential analysis indicates low expression of all these genes in MN patients (Figure 2F,G). Receiver operating characteristic (ROC) curve analysis (Figure 2H–M) revealed that AASS had the highest Area under the curve (AUC) values in both glomeruli and renal tubules. AASS encodes a bifunctional enzyme involved in the early steps of lysine degradation,^3^, ^4^ and AASS low expression in diabetic nephropathy was found to correlate positively with estimated glomerular filtration rate (eGFR) and negatively with serum creatinine, emphasising its significance in renal disease.^5^

**FIGURE 2:**
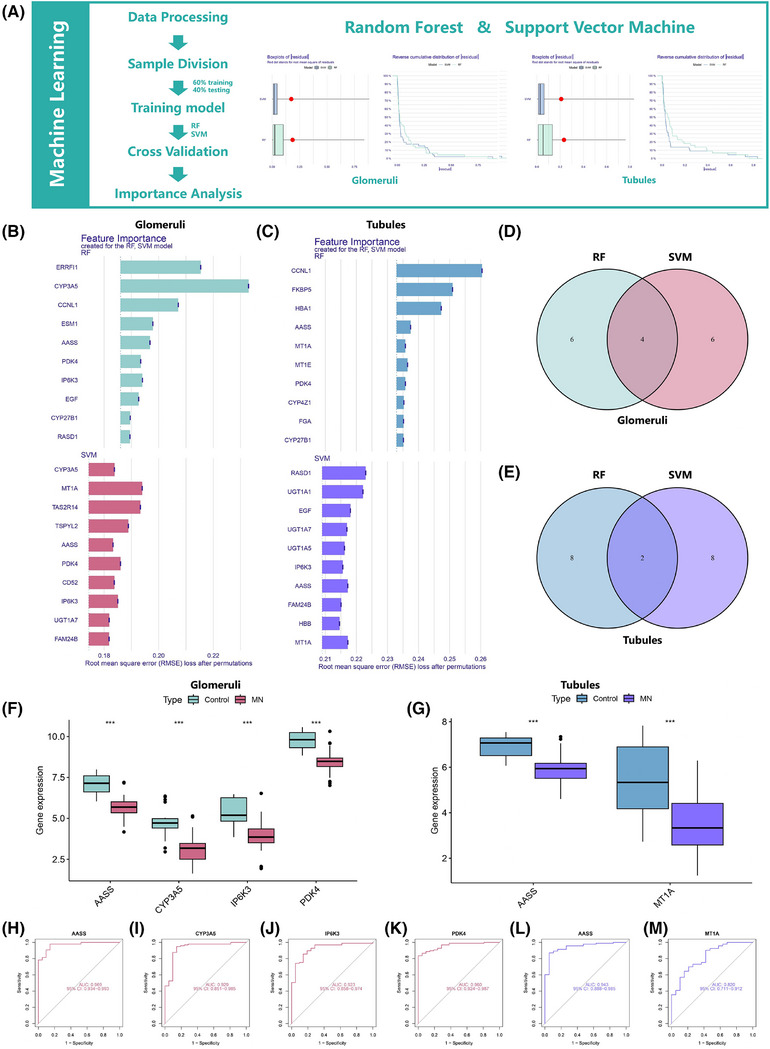
Diagnostic biomarkers screening results for SVM and RF algorithms. (A) The flowchart of this part. (B) Importance ranking plot of variables in glomeruli. (C) Importance ranking plot of variables in renal tubules. (D) Venn diagram of a machine learning algorithm for intersecting genes in glomeruli. (E) Venn diagram of intersecting genes in renal tubules. (F) Difference analysis of diagnostic biomarkers in glomeruli. (G) Boxplot of difference analysis in renal tubules. (H–M) ROC curves of diagnostic biomarkers in glomeruli and renal tubules. RF, random forest; SVM, support vector machine.

Then, we explored the expression of AASS in renal tissue and its correlation with clinical indicators (Figure 3A). Renal tissues were obtained from 40 serum PLA2R‐positive patients diagnosed with MN by renal biopsy at Shengjing Hospital between April and December 2024, following ethical approval and consent procedures. Clinical data were gathered, and patients were divided into impaired renal function (IRF group, eGFR < 60 mL/min/1.73 m^2^) and non‐impaired renal function (non‐IRF, eGFR ≥ 60 mL/min/1.73 m^2^) groups. Immunofluorescence staining revealed that AASS was primarily located in the glomeruli capillary loops, mesangium (Figure 3B), and tubular epithelial cells (Figure 3D). It demonstrated that AASS expression was lower in the IRF group (Figure 3C,E). The IRF group showed higher serum creatinine, 24‐h proteinuria, and anti‐PLA2R levels while lower eGFR, albumin, and haemoglobin levels (Table S2). Alongside correlations between AASS expression and clinical indicators, including negative correlations with serum creatinine and positive correlations with eGFR in glomeruli (Figure 3F,G) and renal tubules (Figure 3J,K). Furthermore, AASS expression in glomeruli (Figure 3H,I) was associated with 24‐h proteinuriaand albumin levels.

**FIGURE 3:**
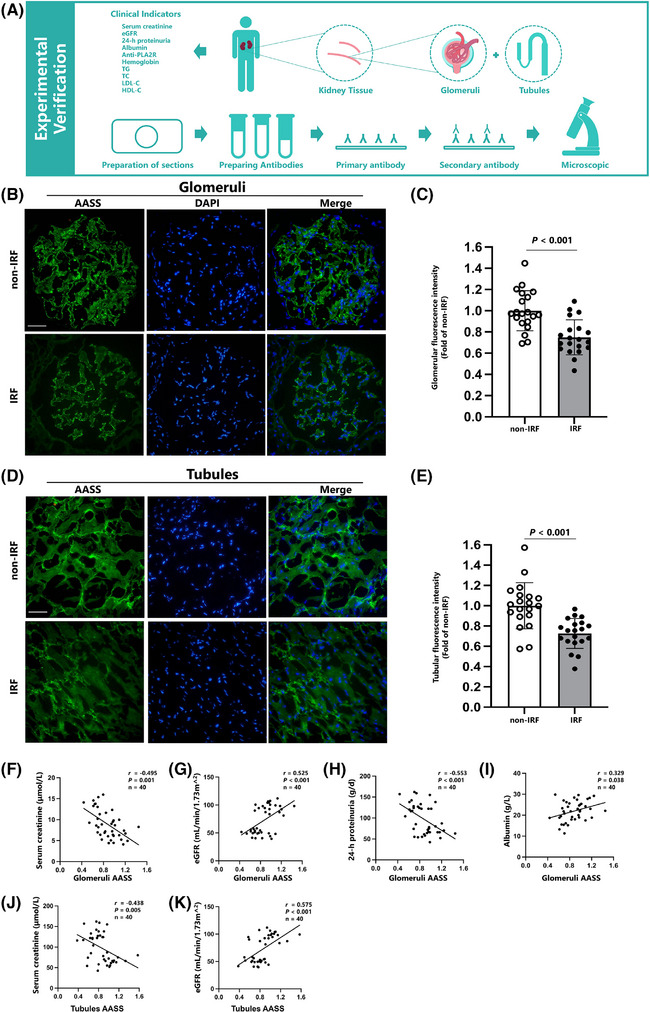
Immunofluorescence of AASS. (A) The flowchart of this part. (B) Immunofluorescence of AASS in glomeruli (magnification × 400, bar  =  20 µm). (C) The semi‐quantification of AASS expression in glomeruli between different groups of MN patients. (D,E) Correlation between AASS and serum creatinine and eGFR. (F) Immunofluorescence of AASS in renal tubules (magnification × 400, bar  =  20 µm). (G) The semi‐quantification of AASS expression in tubules between different groups of MN patients. (H,I) Correlation between AASS and serum creatinine and eGFR in renal tubules. eGFR, estimated glomerular filtration rate; MN, Membranous nephropathy.

Finally, a combination of gene set enrichment analysis (GSEA), Mendelian randomisation (MR), single‐cell RNA sequencing (scRNA‐seq), and molecule docking methods was employed to explore the potential biological mechanisms of AASS in MN (Figure 4A).

**FIGURE 4:**
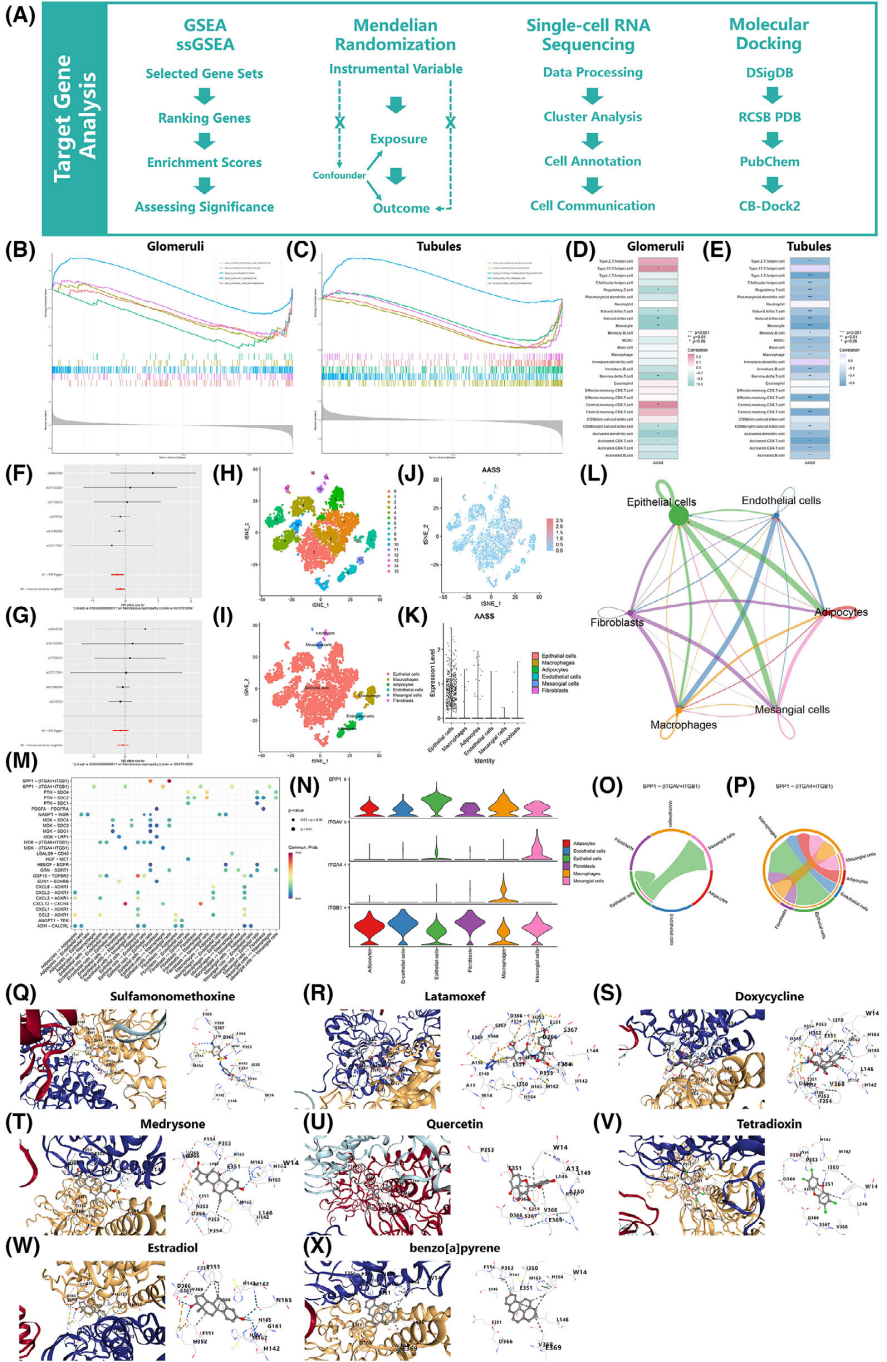
The exploratory study of AASS gene in MN. (A) The flowchart of this part. (B,C) Results of GSEA analysis of AASS genes in glomeruli and renal tubules. (D,E) Heatmap of the correlation between immune cell types and AASS in glomeruli and renal tubules. (F,G) Forest plots of MR analysis [MR Egger and Inverse variance weighted (IVW)] in ebi‐a‐GCST010004 and ebi‐a‐GCST010005. (H) Clustering results of the t‐SNE analysis. (I) Cellular annotation results. (J,K) Expression of AASS in different cells. (L) Cell communication interaction strength maps. (M) Bubble plot of receptor‐ligand pairs. (N) Violin plot of gene expression related to the SPP1 signalling pathway. (O) Chord diagram of SPP1‐(ITGAV+ITGB1). (P) Chord diagram of SPP1‐(ITGA4+ITGB1). (Q–X) Docking patterns of AASS and compounds. MN, Membranous nephropathy; GSEA, gene set enrichment analysis; MR, Mendelian randomisation.

GSEA was conducted utilising KEGG datasets. It indicated that AASS was indirectly associated with systemic lupus erythematosus (SLE) and olfactory transduction pathways in glomeruli and renal tubules (Figure 4B,C). We applied the single‐sample GSEA (ssGSEA) to investigate the profiles of immune cell‐related features. AASS demonstrated predominantly negative correlations with various immune cell types in both glomeruli and renal tubules (Figure 4D,E). The overactive immune system in SLE leads to the production of antibodies against their tissues and may cause MN.^6^ The role of AASS in these pathological processes deserves further study.

The MR study^7^ utilised SNPs as instrumental variables from the GWAS database to explore the association between the exposure factor AASS (eqtl‐a‐ENSG00000008311) and its relationship with MN across different populations (East Asia: ebi‐a‐GCST010004; Europe: ebi‐a‐GCST010005). The analysis indicated (Figure 4F,G) that AASS may serve as a protective factor against MN in East Asian population [Odds ratio (OR) = 0.852, 95% Confidence interval (CI): 0.738–0.985, *P *= 0.030], while no association within European population (Table S3).

We analysed the scRNA‐seq dataset (GSE171458), clustering cells into 16 groups using t‐SNE (Figure 4H) and classifying cell types with the single package (Figure 4I). It revealed that the AASS gene was predominantly expressed in epithelial cells (Figure 4J,K). Additionally, the CellChat package was used to assess intercellular communication (Figure 4L). The SPP1‐(ITGAV+ITGB1) receptor–ligand pair has the highest communication probability (Figure 4M). We focused on the SPP1 signalling pathway, finding high expression of SPP1 and ITGB1 (Figure 4N). The intercellular signalling processes in SPP1‐(ITGAV+ITGB1) and SPP1‐(ITGA4+ITGB1) are different (Figure 4O,P). A study demonstrated that SPP1 interacts with integrins (ITGAV and ITGB1) to promote intercellular signal transduction.^8^ We speculate that AASS may be associated with this pathway, which needs further study.

This study queried the AASS gene from the DSigDB database and retrieved their 3D structures from PubChem, followed by molecular docking using CB‐Dock2 (Table S4). Compounds with a Vina score < −7.0 were selected for further evaluation, including sulfamonomethoxine, latamoxef, doxycycline, medrysone, quercetin, tetradioxin, estradiol and benzo[a]pyrene (Figure 4Q–X). Notably, quercetin, a natural flavonoid, exhibits antioxidant properties that may mitigate kidney damage and enhance long‐term outcomes through various pathways.^9^ Additionally, estradiol, a steroid estrogen, has shown potential in ameliorating ischemia‐reperfusion‐induced kidney injury.^10^

In summary, we analysed the transcriptomic data of glomeruli and renal tubules using machine learning methods. Combining MR, GSEA, ssGSEA, scRNA‐seq analysis, molecular docking, and experimental validation, this study provides a solid foundation for understanding the relationship between AASS and MN.

## AUTHOR CONTRIBUTIONS

Congcong Jiao, Yuxin Zhao and Xu Yang performed the renal tissue puncture, analysed the data using bioinformatics methods and significantly contributed to the manuscript writing. Yang Shao and Haoshen Feng analysed the data using bioinformatics methods, optimised the graphs and tables, and revised the manuscript. Haoshen Feng participated in writing the manuscript and analysing clinical data. Cong Ma and Xiangnan Hao conducted the immunofluorescence staining and analysed clinical data. Xiaomei Liu and Hua Zhou offered expert knowledge for the diagnosis of pathologies. Junjun Luan participated in writing the manuscript and verifying the data. Junjun Luan and Hua Zhou provided financial support and conducted the final review of the manuscript. All authors reviewed and approved the final manuscript.

## CONFLICT OF INTEREST STATEMENT

The authors declare no conflicts of interest.

## FUNDING INFORMATION

This research was supported by Chinese Nature Science Foundation 82170740 (HZ), 82100743 (JJL), Applied Basic Research Program of Liaoning Province 2022JH2/101300048 (HZ), Liao Ning Revitalisation Talents Program XLYC2002081 (HZ), Pandeng Scholar of Liaoning Province 2013222 (HZ) and Outstanding Scientific Fund of Shengjing Hospital of China Medical University 202206 (HZ).

## ETHICS APPROVAL AND CONSENT TO PARTICIPATE

The studies involving human participants were reviewed and approved by the Institutional Review Board of Shengjing Hospital of China Medical University (2024PS760K, 2024‐3‐7). The patients/participants provided their written informed consent to participate in this study.

## Supporting information



Supporting information

Supporting information

## Data Availability

The datasets used and analysed during this study are available from the corresponding author on reasonable request.
