# Supplementary material for: Exploring glomeruli and renal tubules transcriptomic data: Crucial role of the AASS gene in membranous nephropathy
Source: Clin Transl Med. 2025 Apr 23;15(4):e70317. doi: 10.1002/ctm2.70317 (PMC12017891; doi:10.1002/ctm2.70317)

Figure S1: Diagnostic biomarkers screening results for SVM and RF algorithms. **A**: Residual boxplot of machine learning algorithms in glomeruli. **B**: ROC of RF model test set in glomeruli. **C**: Cumulative distribution plot of residuals in glomeruli. **D**: ROC of SVM model test set in glomeruli. **E**: Residual boxplot of machine learning algorithms in renal tubules. **F**: ROC of RF model test set in renal tubules. **G**: Cumulative distribution plot of residuals in renal tubules. **H**: ROC of SVM model test set in renal tubules.


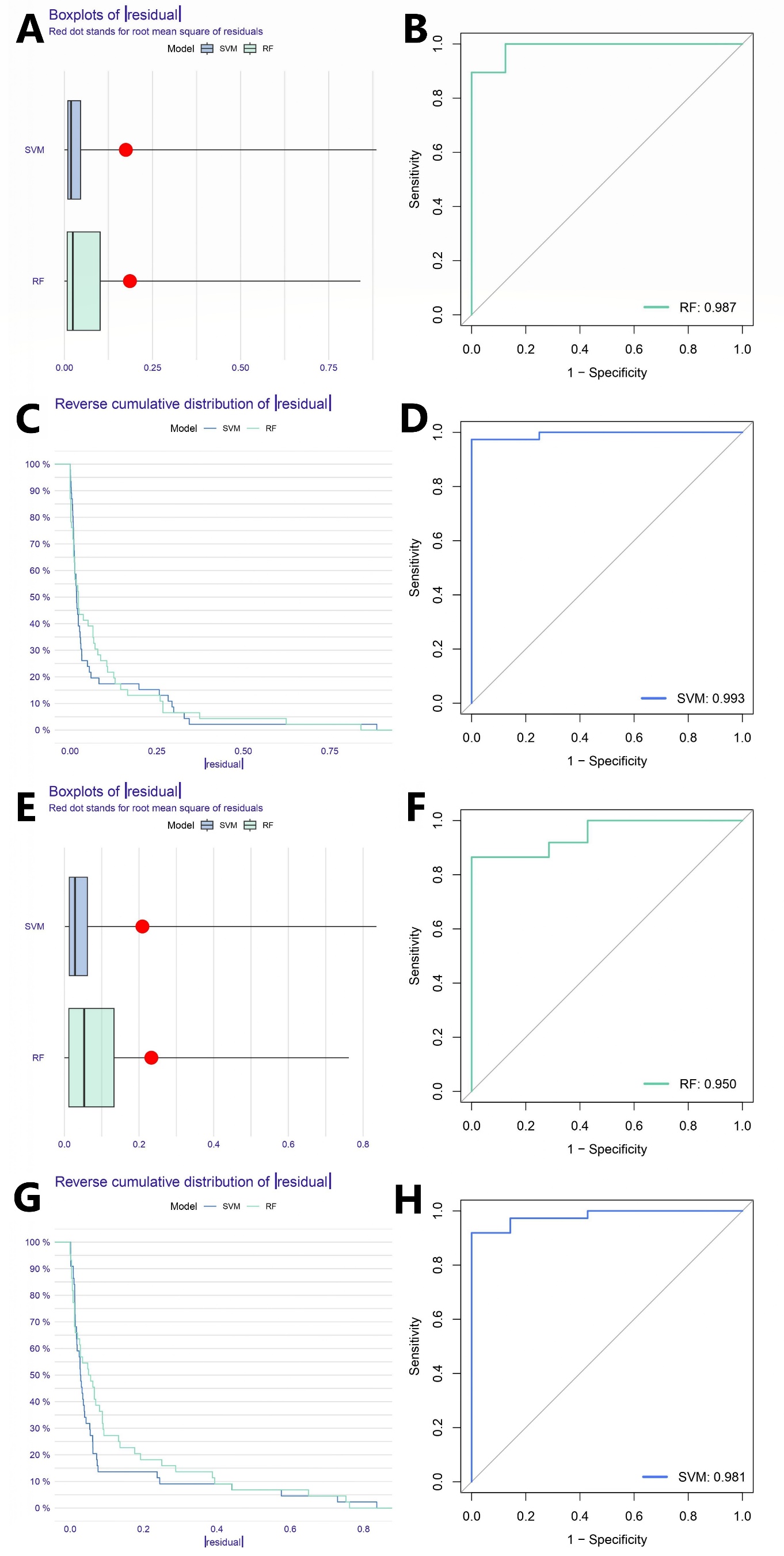

Supplement: Supplementary file 1 — Supporting information [file CTM2-15-e70317-s002.docx]
