# Supplementary material for: Exploring glomeruli and renal tubules transcriptomic data: Crucial role of the AASS gene in membranous nephropathy
Source: Clin Transl Med. 2025 Apr 23;15(4):e70317. doi: 10.1002/ctm2.70317 (PMC12017891; doi:10.1002/ctm2.70317)
Supplement: Supplementary file 2 — Supporting information [file CTM2-15-e70317-s001.docx]

**Table S1:** Information on RNA transcription datasets in GEO.

| **GEO id** | **Platform** | **Type** | **MN** | **Control** |
| --- | --- | --- | --- | --- |
| GSE108109 | GPL19983 | Glomeruli | 44 | 6 |
| GSE108112 | GPL19983 | Renal tubule | 43 | 5 |
| GSE180393 | GPL19983 | Glomeruli | 2 | 9 |
| GSE180394 | GPL19983 | Renal tubule | 2 | 9 |
| GSE200818 | GPL19983 | Renal tubule | 48 | 5 |
| GSE200828 | GPL19983 | Glomeruli | 51 | 6 |

| **Clinical indicators** | **non-IRF** | **IRF** | ***P-*value** |
| --- | --- | --- | --- |
| Serum creatinine (μmol/L) | 65.27 ± 2.41 | 129.75 ± 4.27 | **< 0.001** |
| eGFR (mL/min/1.73m^^2^) | 96.59 ± 1.83 | 49.41 ± 1.27 | **< 0.001** |
| 24-h proteinuria (g/d) | 7.20 ± 0.63 | 10.62 ± 0.68 | **< 0.001** |
| Albumin (g/L) | 23.71 ± 0.90 | 20.10 ± 1.10 | **0.014** |
| Anti-PLA2R (RU/mL) | 46.86 ± 4.43 | 109.56 ± 14.26 | **< 0.001** |
| Hemoglobin (g/L) | 136.15 ± 2.54 | 121.20 ± 5.25 | **0.014** |
| TG (mmol/L) | 3.26 ± 0.45 | 2.97 ± 0.37 | 0.622 |
| TC (mmol/L) | 7.10 ± 0.48 | 7.76 ± 0.61 | 0.239 |
| LDL-C (mmol/L) | 4.06 ± 0.35 | 4.58 ± 0.52 | 0.407 |
| HDL-C (mmol/L) | 1.35 ± 0.10 | 1.21 ± 0.08 | 0.508 |

**Table S2**: Details of clinical indicators related to MN patients.

**Table S3:** MR results of AASS genes with MN datasets.

| Outcome | Method | β | Se | *P* | OR  (95% CI) | Heterogeneity *P*-value | Pleiotropy *P*-value |
| --- | --- | --- | --- | --- | --- | --- | --- |
| ebi-a-GCST010004 | MR Egger | -0.243 | 0.100 | 0.073 | 0.784 (0.644~0.955) | 0.854 | 0.289 |
| ebi-a-GCST010004 | Weighted median | -0.176 | 0.078 | **0.024** | **0.839 (0.720~0.977)** | - |  |
| ebi-a-GCST010004 | IVW | -0.160 | 0.074 | **0.030** | **0.852 (0.738~0.985)** | 0.726 |  |
| ebi-a-GCST010004 | Simple mode | -0.098 | 0.151 | 0.544 | 0.907 (0.675~1.218) | - |  |
| ebi-a-GCST010004 | Weighted mode | -0.179 | 0.079 | 0.074 | 0.836 (0.715~0.977) | - |  |
| ebi-a-GCST010005 | MR Egger | -0.139 | 0.128 | 0.339 | 0.870 (0.677~1.119) | 0.966 | 0.464 |
| ebi-a-GCST010005 | Weighted median | -0.086 | 0.098 | 0.380 | 0.918 (0.758~1.111) | - |  |
| ebi-a-GCST010005 | IVW | -0.069 | 0.094 | 0.466 | 0.933 (0.776~1.123) | 0.942 |  |
| ebi-a-GCST010005 | Simple mode | 0.036 | 0.182 | 0.852 | 1.036 (0.726~1.480) | - |  |
| ebi-a-GCST010005 | Weighted mode | -0.087 | 0.102 | 0.433 | 0.916 (0.750~1.120) | - |  |

**Table S4:** Docking of AASS with its related compounds in DSigDB.

| ID | PubChem CID | CurPocket ID | Vina score |
| --- | --- | --- | --- |
| N-acetyl-L-aspartic acid | 65065 | C4 | -5.3 |
| Sulfamonomethoxine | 5332 | C2 | **-7.0** |
| Latamoxef | 47499 | C2 | **-8.7** |
| Doxycycline | 54671203 | C2 | **-9.2** |
| Anisomycin | 253602 | C3 | -6.7 |
| Medrysone | 247839 | C2 | **-9.5** |
| ZINC | 23994 | NA | NA |
| Quercetin | 5280343 | C3 | **-8.7** |
| SELENIUM | 6326970 | NA | NA |
| Tetradioxin | 15625 | C2 | **-7.3** |
| Hydrogen peroxide | 784 | C3 | -2.9 |
| Estradiol | 5757 | C2 | **-8.7** |
| VITAMIN E | NA | NA | NA |
| benzo[a]pyrene | 2336 | C2 | **-9.7** |
| Acetaminophen | 1983 | C5 | -5.6 |
| 7646-79-9 | 24288 | NA | NA |
| cyclosporin A | 5284373 | NA | NA |
| VALPROIC ACID | 3121 | C2 | -4.9 |

*ZINC, SELENIUM, VITAMIN E, 7646-79-9, and cyclosporin A lacked corresponding 3D structures in PubChem database.
